# Prevalence, demographic and spatial distribution of treated epilepsy in France in 2020: a study based on the French national health data system

**DOI:** 10.1007/s00415-023-11953-2

**Published:** 2023-10-03

**Authors:** Joël Coste, Laurence Mandereau-Bruno, Laure Carcaillon-Bentata, Yann Mikaeloff, Viviane Bouilleret

**Affiliations:** 1 Santé Publique France (French National Public Health Agency), Saint-Maurice, France; 2 CPEA, Assistance Publique-Hôpitaux de Paris, Groupement Hospitalo-Universitaire Paris-Saclay, Paris-Saclay University, Paris, France; 3 CESP-INSERM, Le Kremlin-Bicêtre, France; 4 Neurophysiology and Epileptology Department, Hôpital Bicêtre, Assistance Publique des Hôpitaux de Paris (AP-HP), Hôpitaux Universitaires Paris-Saclay, Le Kremlin-Bicêtre, France; 5 Laboratoire d’Imagerie Biomédicale Multimodale (BioMaps), Service Hospitalier Frédéric Joliot, CEA, CNRS, Inserm, Université Paris-Saclay, Orsay, France; 6 School of Medicine, Université Paris Saclay, Le Kremlin Bicêtre, France

**Keywords:** Epilepsy, Prevalence, Socio-economic deprivation, Territorial heterogeneity, Health claims database

## Abstract

**Background:**

Although still incomplete, the epidemiology of epilepsy shows substantial variations in the burden of the condition according to demographic, social and territorial characteristics. This study aimed to estimate the prevalence of treated epilepsy and to investigate its demographic and spatial distribution in 2020 in France, a country where the nationwide epidemiological situation of the condition remains largely unknown.

**Methods:**

We used the French national health data system, which covers nearly the entire population residing in France (over 67 million of inhabitants in metropolitan and overseas departments). Prevalent cases were identified using long-term disease status, hospitalisation for epilepsy (ICD-10 codes G40 or G41), and reimbursements for antiseizure medications and electroencephalograms.

**Results:**

In 2020, we identified 685,122 epilepsy cases, corresponding to an overall prevalence of 10.2 per 1000 inhabitants [95% confidence interval 10.1–10.2], with similar rates in men and women. Estimates were found to increase with age, with an accelerated rise in the second half of the life, which occurred earlier in men than in women. We observed a monotonic gradient of variation with socio-economic deprivation (in non-military metropolitan subjects aged 18–54 years) as well as territorial heterogeneity, with the mountainous centre of France as well as some French overseas departments having the highest prevalence.

**Conclusions:**

Our results revise upwards the estimation of epilepsy prevalence in France, showing that it now ranks among the highest in developed countries. Our study also confirms the important socio-territorial heterogeneity of the condition that reflects health inequalities in this country.

**Supplementary Information:**

The online version contains supplementary material available at 10.1007/s00415-023-11953-2.

## Introduction

Epilepsy is a common and serious disorder [1], but its epidemiology is still incomplete. Indeed, epidemiological investigations have been hampered by differences in study methodology and definitions of active epilepsy [2]. Aside from age and sex, socio-economic and income levels have been identified as the most important factors affecting the incidence and prevalence of epilepsy around the world [3, 4]. Even in high-income countries, the burden of epilepsy appears to be greater in socio-economically deprived populations [5–8]. However, social and territorial variability are seldom explored in large nationwide studies of active epilepsy [6, 9].

Medico-administrative or health claims databases allow for the epidemiological investigation of many diseases [10]. To date, several neurological diseases, including multiple sclerosis, Parkinson’s disease and dementia, have been examined in various prevalence or incidence studies (e.g. [11–16]). Epilepsy has also been investigated in some of these studies conducted over the last decade, most of which consider treated epilepsy [5, 17–21], which can be easily assessed in claims databases [19].

The present study aimed to estimate the prevalence of treated epilepsy in France (metropolitan and overseas departments) and to investigate its demographic and spatial distribution in 2020 using the French national health data system. In this country characterised by substantial health inequalities, the nationwide epidemiological situation of the condition is largely unknown, especially in children and older adults. One study conducted in 1995 reported the prevalence of epilepsy (active or not, treated or not) in subjects aged over 15 years in a medium-sized city of southern France [22]. Another study was carried out on a subsample (1/97) of the national health database in 2009, but it only considered subjects over 16 years undergoing polytherapy with at least two antiseizure medications [23].

## Materials and methods

### Data sources

This study used data from the French national health data system or *Système National des Données de Santé* (SNDS) [24]. The SNDS includes several databases with individualised, pseudo-anonymised and comprehensive healthcare data for the beneficiaries of the different health insurance schemes in France. The SNDS covers nearly the entire population residing in France (67,162,154 inhabitants on 1 January 2020). Data include all medication reimbursements, laboratory tests, paraclinical investigations and medical information on hospitalisations in public and private hospitals (outpatient admission, rehabilitation, psychiatry or home care services) since 2006. Medications are coded using the Anatomical Therapeutic Chemical (ATC) Classification System, while laboratory tests and paraclinical investigations are coded using a standardised coding system of clinical procedures known as the *Classification Commune des Actes Médicaux* (CCAM). For each hospital stay, the admission and discharge dates are recorded along with several diagnostic codes using the International Classification of Diseases-10th Revision (ICD-10). Individuals with long-term chronic diseases (LTDs), including epilepsy, who benefit from free healthcare are also recorded and coded using the ICD-10. Demographic data, namely age, sex, place of residence and deprivation index of the place of residence (Fdep index), are also available. The Fdep index is constructed using median household income, proportion of secondary school graduates among inhabitants aged 15 years and over, proportion of manual labourers in the active population and proportion of unemployment in the place of residence [25]. Note that Fdep can only be calculated for non-military metropolitan subjects. By contrast, the following information is absent from the SNDS: socio-economic characteristics such as marital status, employment and type of job; risk factors such as smoking, alcohol and lifestyle; clinical examination findings such as blood pressure and body mass index; results from laboratory tests and paraclinical investigations; and reasons for medical or paramedical consultation, including hospital outpatient consultations.

### Identification of subjects with epilepsy

Several data sources were used to identify prevalent treated epilepsy, excluding occasional treatments such as those used during cerebral neurosurgical interventions: reimbursements for an antiseizure medication (ASM) or electroencephalogram (EEG), LTD for epilepsy and hospitalisation for epilepsy.

A subject was considered to have treated epilepsy based on the presence of at least one of the following criteria over a 5-year period (2015–2019) (subjects meeting more than one criteria were counted only once):

1. LTD for epilepsy (ICD-10 codes G40 or G41) with at least one reimbursement of an ASM in the same year (ATC code N03A except for Valpromide N03AG02);
2. Hospitalisation (outpatient admission, rehabilitation, psychiatry or home care services) with a diagnostic code for epilepsy (same ICD-10 codes as above) followed by the reimbursement of an ASM (same ATC codes as above) within 3 months of hospitalisation;
3. At least three reimbursements of an ASM (same ATC codes as above) at three different dates in the same year and zero to two reimbursements of an EEG (at different dates in the same year) depending on the prescription “specificity” of the ASM. When an ASM is exclusively or mostly used for epilepsy (e.g. phenobarbital, levetiracetam, topiramate), we considered it useless or even disadvantageous to require an EEG for case identification. On the contrary, when an ASM is massively prescribed for non-epileptic subjects, especially for chronic pain (e.g. gabapentin, pregabalin, clonazepam), two EEGs would be necessary to retain the case. In intermediate cases, for ASMs such as carbamazepine or lamotrigine that are also commonly prescribed in psychiatry, one EEG was required (detailed results of the case-by-case analysis are given in Supplementary Table 1).

In the absence of consensual recommendations for EEG use in the follow-up of patients treated for epilepsy, the reimbursement of an EEG was not considered mandatory in our algorithm except when combined with ASMs which can also be used as painkillers or for psychiatric disorders.

### Statistical analysis

The prevalence of epilepsy was defined as the number of epilepsy cases identified on 1 January 2020 divided by the number of people residing in France (including the overseas departments except for Mayotte) on this date. Strata of sex and age (5-year intervals) were considered. Geographical variations were studied using standardised prevalence values by “department” (third level of the Nomenclature of Territorial Units for Statistics [NUTS 3], *N* = 100). The reference population for standardisation was the general population residing in France (except for Mayotte) on 1 January 2020. For people aged 18–54 years (not yet affected by cardiovascular comorbidities, which have well-known social determinants), variations in standardised prevalence were also investigated by quintiles of the Fdep index based on the place of residence.

## Results

### Description of the population

On 1 January 2020, a total of 685,122 patients with epilepsy were identified in France based on the SNDS, with 45% of these patients being identified by at least two selection criteria. Among all epilepsy cases, 86% had ASM with/without EEG reimbursements, 41% underwent hospitalisation and 29% had LTD status. In addition, 43% of epilepsy cases were only identified by ASM with/without EEG reimbursements, not by hospitalisation or LTD status. The sex ratio (female/male) was 1.07.

### Prevalence and demographic differences

**Fig. 1:**
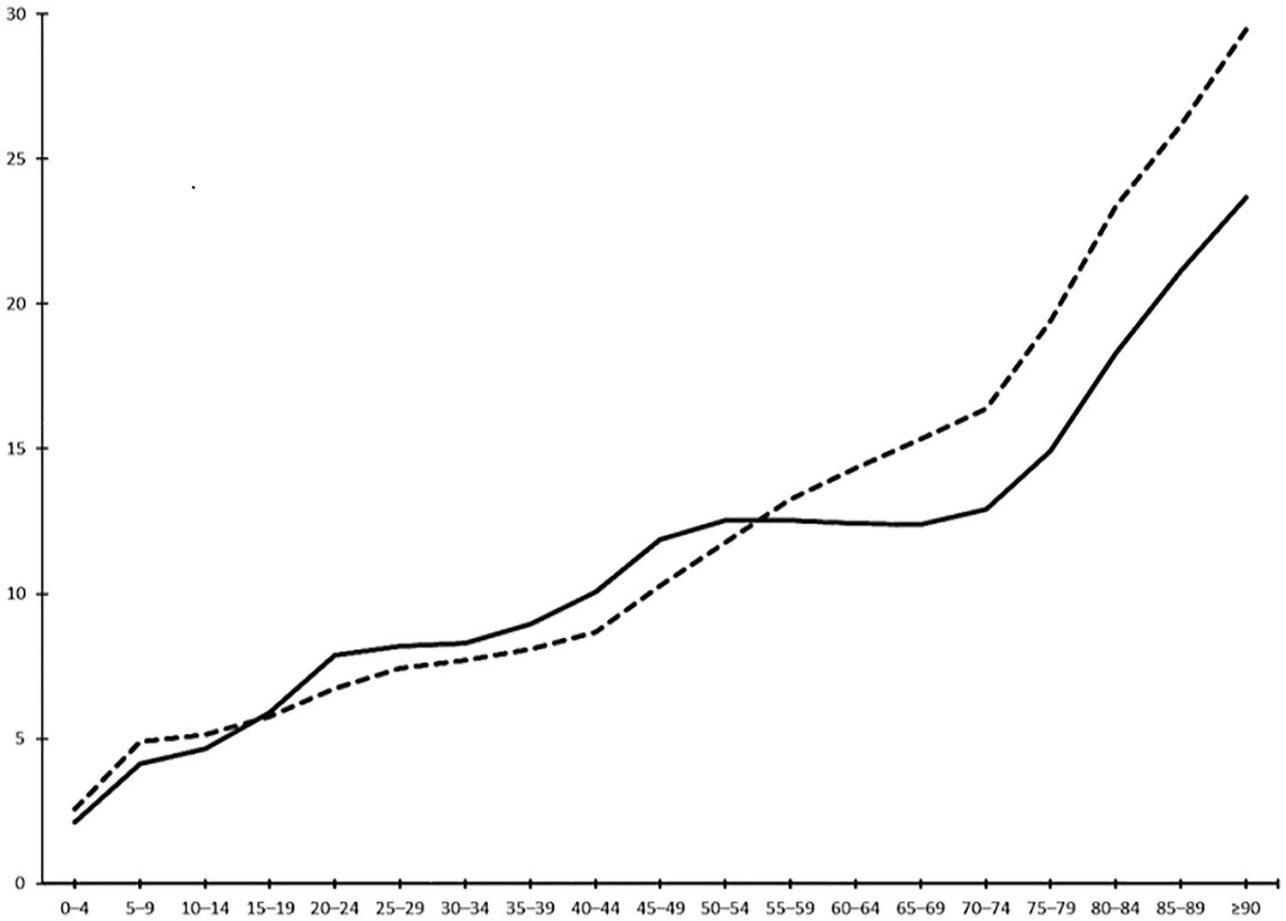
Prevalence (per 1000) by sex and age group (men: dotted line, women: solid black line). *X*-axis: age group, *Y*-axis: prevalence per 1000 inhabitants

On 1 January 2020, the prevalence of epilepsy in France was 10.2 per 1000 inhabitants (95% confidence interval 10.1–10.2), being the same for both men and women. The prevalence by sex and age groups is provided in Table 1 and Fig. 1. In men, prevalence increases gradually from 2.6 to 8.7 per 1000 inhabitants at 40–44 years and then more steeply, especially after 65–69 years, reaching 20 per 1000 inhabitants at 80 years. In women, the initial trend is similar, with slightly higher values than in men before reaching a plateau of around 12 per 1000 inhabitants between 45 and 74 years and then rising steeply as in men.

**Table 1:** Prevalence (per 1000) of epilepsy in France on 1 January 2020

Age (years)	Overall			Men			Women		
	Number of cases	Population	Prevalence	Number of cases	Population	Prevalence	Number of cases	Population	Prevalence
0–4	8567	3,636,987	2.4	4789	1,860,219	2.6	3778	1,776,768	2.1
5–9	18,503	4,080,868	4.5	10,232	2,085,359	4.9	8271	1,995,509	4.1
10–14	20,523	4,191,412	4.9	11,030	2,146,447	5.1	9493	2,044,965	4.6
15–19	24,263	4,153,672	5.8	12,326	2,137,400	5.8	11,937	2,016,272	5.9
20–24	27,584	3,781,203	7.3	12,929	1,922,546	6.7	14,655	1,858,657	7.9
25–29	29,346	3,751,382	7.8	13,828	1,862,011	7.4	15,518	1,889,371	8.2
30–34	32,603	4,071,149	8.0	15,276	1,982,648	7.7	17,327	2,088,501	8.3
35–39	36,162	4,230,660	8.5	16,722	2,061,745	8.1	19,440	2,168,915	9.0
40–44	38,304	4,078,159	9.4	17,455	2,006,632	8.7	20,849	2,071,527	10.1
45–49	50,138	4,520,870	11.1	23,029	2,238,088	10.3	27,109	2,282,782	11.9
50–54	53,927	4,435,547	12.2	25,665	2,178,912	11.8	28,262	2,256,635	12.5
55–59	56,469	4,378,191	12.9	28,244	2,127,193	13.3	28,225	2,250,998	12.5
60–64	54,914	4,115,711	13.3	28,141	1,962,928	14.3	26,773	2,152,783	12.4
65–69	53,862	3,908,689	13.8	28,215	1,838,196	15.3	25,647	2,070,493	12.4
70–74	50,597	3,484,027	14.5	26,431	1,612,913	16.4	24,166	1,871,114	12.9
75–79	37,514	2,216,024	16.9	19,157	987,690	19.4	18,357	1,228,334	14.9
80–84	38,175	1,874,175	20.4	17,902	766,269	23.4	20,273	1,107,906	18.3
85–89	31,419	1,370,243	22.9	12,691	485,017	26.2	18,728	885,226	21.2
≥ 90	22,252	883,185	25.2	6901	234,287	29.5	15,351	648,898	23.7
Total	685,122	67,162,154	10.2	330,963	32,496,500	10.2	354,159	34,665,654	10.2

### Spatial distribution

**Fig. 2:**
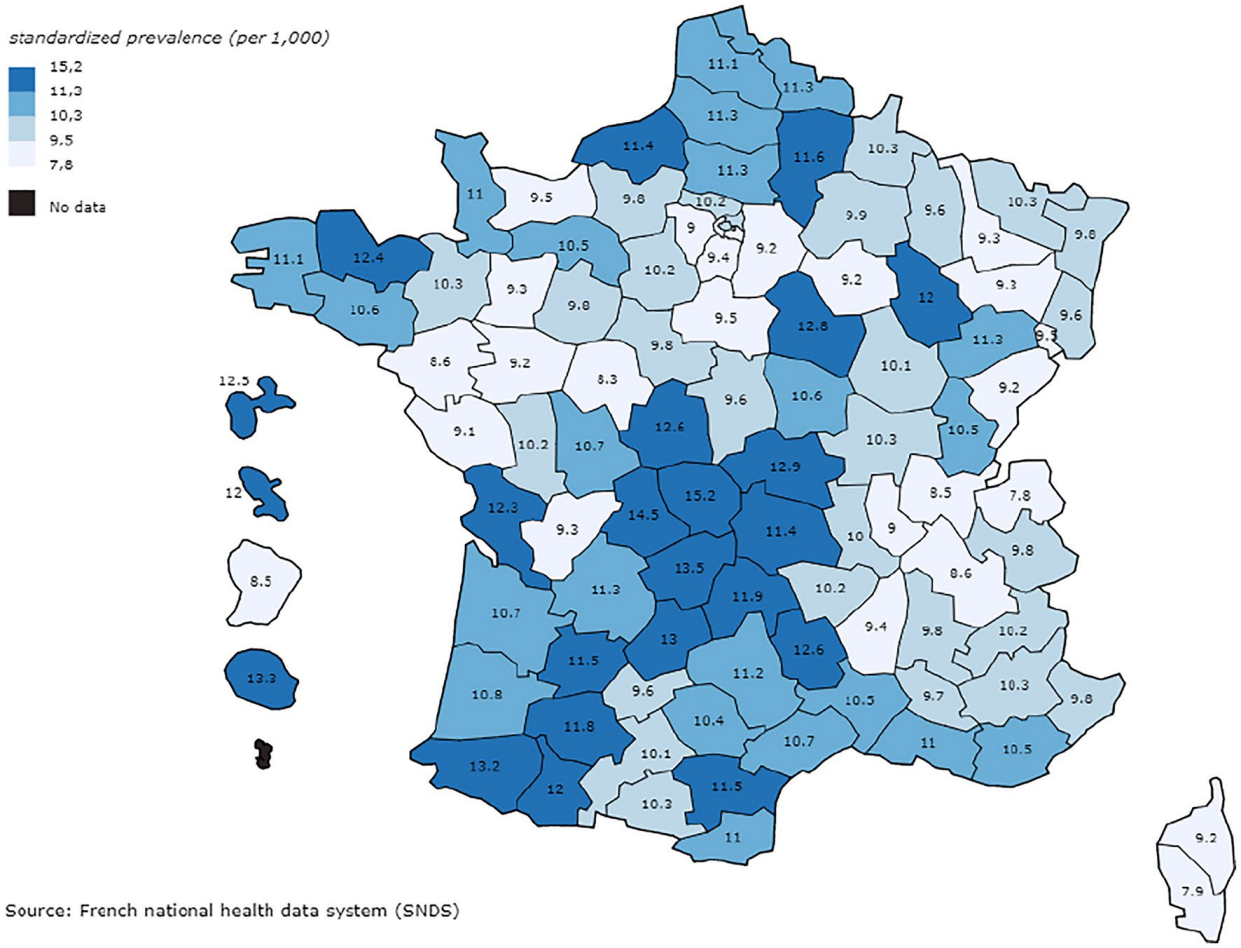
Standardised prevalence (per 1000 inhabitants) of epilepsy at the department level in France on 1 January 2020

The standardised prevalence by department is illustrated in Fig. 2. The standardised prevalence estimates were higher in the northern departments (Nord, Aisne) and in those along the north-east to south-west diagonal, including departments in the mountainous centre of France. The standardised prevalence estimates in the French overseas departments were generally high, exceeding 12 cases per 1000 inhabitants, except in Guyana (8.5 per 1000 inhabitants).

When considering the level of socio-economic deprivation according to the place of residence, a monotonic gradient of variation was found, with 42% excess of subjects living in the most deprived quintile (10.1 cases per 1000 inhabitants) compared with those living in the least deprived quintile (7.1 cases) (Supplementary Table 2).

## Discussion

To our knowledge, this is the first study to provide the prevalence estimates of treated epilepsy at the national and departmental levels in France as well as one of the largest studies on the national prevalence of treated epilepsy ever conducted in the world.

### Overall prevalence

This study found an all-age prevalence of treated epilepsy of 10.2 cases per 1000 inhabitants, which is much higher than previous figures reported in France for specific populations (i.e. medium-size city in southern France, polytherapy) [22, 23]. Our results are also much higher than those observed in Korea in 2009 [19] (3.8 cases per 1000 inhabitants), in Japan in 2019 (6.0 per 1000 inhabitants) [21] and in the European Union in 2017 by the Global Burden of Disease Study (3.6 per 1000 inhabitants) [7]. They are slightly higher than those found in England in 2007–08 (8.0 per 1000 inhabitants) [Steer], in the USA for the period 2007–11 (8.5 per 1000 inhabitants) [17] and in the UK in 2019 (9.4 per 1000) [8]. Nevertheless, when considering comparable age intervals, our figures are about 25% lower than those found in the study of Kariboom et al. on Americans aged 18–64 years with poor health and low incomes for the period 1992–2006 [5]. Using a slightly different methodology to estimate “active epilepsy” (self-reported history of doctor-diagnosed epilepsy and ASM consumption), Zack et al. observed a slightly higher prevalence of 12 cases per 1000 inhabitants in the USA in 2015 [9].

### Prevalence according to age

Regarding the age distribution, the steady and continual rise in the prevalence of epilepsy until midlife has likewise been observed in the USA [15, 16]. Contrary to incidence, which exhibits a U or J shape due to high values in the first year of life, prevalence, due to its mathematical relation to incidence, rises progressively or is steady throughout childhood [17, 18]. However, the prevalence observed in this study for the paediatric population (4.5 per 1000 inhabitants for 0–19 years) is slightly lower than that observed in the USA in 2012 by Kim et al. (6.8 per 1000 inhabitants) [18]. The recent evolution of the therapeutic guidelines for young children in whom no treatment is recommended for benign forms of epilepsy may also partly explain these results. The accelerated rise in epilepsy in the second half of the life, earlier in men than in women, was also observed in the USA and Japan [5, 21] as well as in several studies based on survey data [4, 7]. This result may be explained by the different prescription strategies, since in the young population, the potential side effects of medications means that their duration of use is limited, especially because some forms of epilepsy can disappear with age. Conversely, in older patients, the risk, for example, of a traumatic fall in the event of seizures can result in the continuation of treatment. Despite a consensus on treatment initiations [26], no study to date has explored therapeutic interruption. The increase in epilepsy prevalence at an earlier age in men can mainly be explained by comorbidities, especially cardiovascular disorders, in particular stroke, which is a cause of epilepsy [27] and increases with age and occurs more severely and earlier in men than in women [28, 29].

### Prevalence according to sex

Regarding the sex ratio (female/male), our result (1.07) contradicts many previous studies that reported a slight predominance of men among subjects with epilepsy [1]. This may be explained, especially between 20 and 54 years, by the lower treatment delivery in men than in women due to poorer treatment compliance, which has already been demonstrated in this age group [30]. Another explanation for our finding may be the more frequent occurrence of some idiopathic generalised epilepsies in women, with a typical onset during adolescence as in the case of juvenile myoclonic epilepsy. Due to the medication dependence of these conditions, treatment maintenance is recommended. From the age of 50–54 years onwards, the incidence for both sexes then converges before a male excess in the prevalence of epilepsy emerges in line with the greater risk of lesional epilepsy in older men. Likewise, the sex differences observed in our study are possibly linked to the frequent causes of epilepsy in later life such as traumatic brain injury and stroke, which have a greater incidence in men [28, 29].

### Prevalence according to place of residence and social deprivation

This study revealed notable territorial and social heterogeneity in the prevalence of epilepsy in France. In the overseas departments, the prevalence of epilepsy is contrasted, with the highest rates in Réunion Island and the lowest in Guyana where limited healthcare access may be reflected in the lower prevalence based on the SNDS. The findings for metropolitan France are also heterogeneous, with some departments in northern and central France having a higher prevalence, probably because of the frequency of cardiovascular comorbidities and socio-economic deprivation in these departments [31]. The striking observation of a dose–response gradient of prevalence with social deprivation in France has similarly been observed in other countries and healthcare systems, irrespective of age [5, 6, 8, 18, 20]. Evidence is accumulating that the relationship between epilepsy and social deprivation is causally bidirectional. On the one hand, stigmatisation, treatment side effects, comorbidities, especially psychiatric disorders, and low self-efficacy in epileptic subjects have been associated with greater difficulty of finding and maintaining employment, thus leading to income deprivation [32, 33]. On the other hand, the increased incidence of first seizures observed in socially deprived subjects [34, 35] suggests that genetic and developmental (including *intra utero* and early childhood noxious exposure) or environmental (pollution) factors may be mediators in the relationship.

### Study strengths and limitations

The main strength of our study is its use of a large nationwide dataset, which provides prevalence values according to the main demographic and territorial characteristics of France using the same methodology and identification algorithms. The main limitations are its use of treated epilepsy as a proxy of “active” epilepsy and the inability to identify clinical subtypes, although this is typical of health claims databases. However, the usage of “active” or more commonly treated epilepsy is common in epidemiological studies and is not without relevance. Indeed, anything that disturbs the normal pattern of neuronal activity—from illness to brain damage or abnormal brain development—can trigger seizures. Moreover, epilepsy must be distinguished from provoked seizures caused by a known transient precipitating factor such as high fever, nervous system infection, acute traumatic brain injury or fluctuations in blood sugar or electrolyte levels. In the case of provoked seizures, the seizure disappears once the disorder is controlled, unlike epilepsy that is defined by the occurrence of unprovoked seizures. Rare conditions as with benign forms of epilepsy may not be treated [36], although the vast majority of cases of epilepsy require treatments to prevent the recurrence of seizures. Treated epilepsy may, therefore, be assessed based on the use of ASM, which thus strengthens the diagnostic accuracy and avoids the inclusion of provoked seizures in the analysis. Regarding the subtypes of epilepsy, the information present in the French national health data system (SNDS) is too incomplete (as is generally the case with claims databases), which prevented any attempts at sub-categorisation.

The algorithm used in this study, which relies on various prescription patterns of ASM in epilepsy, has not been formally validated with medical records, although the previous development of a similar algorithm in Korea in 2009, also based on ASM prescription and ICD-10 epilepsy codes, lends support to its use here [19]. The differences between the health systems and claims databases as well as the study periods prevent any extrapolation. However, medication deliveries are among the most reliable data in claim databases, notably in the French national health data system [22].

## Conclusion

Based on the French national health data system that covers nearly the entire French population, the prevalence of epilepsy was estimated at 10.2 cases per 1000 inhabitants. This estimation is much higher than previous figures reported in France and comparable to recent American estimations. It is, thus, one of the highest rates in developed countries, which may be of interest to clinicians, researchers and policymakers in all developed countries evaluating the public health impact of epilepsy. The French national health data system appears to be a useful tool for epilepsy surveillance, especially in view of the important social and territorial heterogeneity of its prevalence, which reflects the current health inequalities, a public health priority in France.

### Supplementary Information

Below is the link to the electronic supplementary material.

Supplementary file1 (DOCX 19 KB)
