# Supplementary material for: Prevalence, demographic and spatial distribution of treated epilepsy in France in 2020: a study based on the French national health data system
Source: J Neurol. 2023 Oct 3;271(1):519–25. doi: 10.1007/s00415-023-11953-2 (PMC10770219; doi:10.1007/s00415-023-11953-2)
Supplement: Supplementary file 1 — Supplementary file1 (DOCX 19 KB) [file 415_2023_11953_MOESM1_ESM.docx]

Supplemental Table 1. Antiseizure medication, frequency of prescription, number of electroencephalograms (EEG) requested within a 5-year period for case identification, and rationale for defining the number of EEG

* Proportion of prescriptions of a given ATC class medication during the 5-year period considered in the study

Supplemental Table 2. Prevalence (per 1,000) of epilepsy in France according the level of deprivation of the place of residency*. Only subjects 18-54 years are considered (see Methods section).

|  | Prevalence |
| --- | --- |
| First quintile (least deprived) | 7.1 |
| Second quintile | 7.8 |
| Third quintile | 9.1 |
| Fourth quintile | 9.5 |
| Fifth quintile (most deprived) | 10.1 |

* The deprivation index was calculated for 98.9% of the selected sample (non-military adults aged 18-54 years with at least for one health expenditure reimbursement in 2019)
